# Prognostic value of preoperative systemic inflammatory response as a prognostic indicator in patients with early-stage oral squamous cell carcinoma

**DOI:** 10.1097/MD.0000000000030855

**Published:** 2022-11-04

**Authors:** Toshihiko Mikami, Akinori Funayama, Kanae Niimi, Kenta Haga, Masami Kawaharada, Akihiko Nakamura, Wataru Katagiri, Tadaharu Kobayashi

**Affiliations:** a Division of Reconstructive Surgery for Oral and Maxillofacial Region, Faculty of Dentistry & Graduate School of Medical and Dental Sciences, Niigata University, Niigata, Japan; b Department of Dentistry and Oral Surgery, Niigata Medical Center, Niigata, Japan; c Department of Oral and Maxillofacial Surgery, Gifu University Graduate School of Medicine, Gifu, Japan.

**Keywords:** delayed cervical, lymph node metastasis, lymphocyte-to-monocyte ratio, neutrophil-to-lymphocyte ratio, oral squamous cell carcinoma, platelet-to-lymphocyte ratio

## Abstract

To determine the usefulness of lymphocyte-to-monocyte ratio (LMR), neutrophil-to-lymphocyte ratio (NLR), platelet-to-lymphocyte ratio (PLR), and inflammatory response biomarker (IRB) score for predicting disease-specific survival and delayed cervical lymph node metastasis in early-stage oral squamous cell carcinoma (OSCC). We retrospectively analyzed 72 patients with early-stage OSCC. Receiver operating characteristic curve analysis was used to determine the cutoff values for LMR, NLR, and PLR. IRB score was determined as follows: high LMR, high NLR, and low PLR, which were each rated as 1. These scores were added to obtain IRB score (range: 0–3). From univariate analysis, gender, poor mode of invasion, and high IRB score were identified as significant risk factors for disease-specific survival. However, there were no independent factors for poor prognosis in multivariate analysis. On the other hand, for delayed cervical lymph node metastasis, poor mode of invasion, low LMR, high NLR, high PLR, and high IRB score were identified as significant risk factors from univariate analysis, and in multivariate analysis, poor mode of invasion and high IRB score were confirmed as independent risk factors. IRB score and mode of invasion are potentially independent risk factors for delayed cervical lymph node metastasis in early-stage OSCC.

## 1. Introduction

Inflammation seems to play a critical role in the development and progression of numerous cancers,^[1,2]^ and it has become clear that cancer-associated inflammation, in the form of local and systemic inflammatory responses (SIRs), is a key factor for disease progression and survival in several cancers.^[3,4]^ Recently, scoring systems using SIR biomarkers such as lymphocyte-to-monocyte ratio (LMR), neutrophil-to-lymphocyte ratio (NLR), and platelet-to-lymphocyte ratio (PLR) have been designed to predict the prognosis of patients with various carcinomas^[5–9]^ including oral cancer.^[10–19]^ Furthermore, Hirahara et al^[20]^ proposed a prognostic scoring system using inflammatory response biomarkers that consists of these three values called inflammatory response biomarker (IRB) score for esophageal cancer patients, and they showed that the scoring system is effective for predicting the cause-specific survival of esophageal cancer patients.

Thickness of the tumor, grade of differentiation, pattern of invasion and E-cadherin expression are currently used markers for calculating the risk factors of cervical lymph node metastasis from oral squamous cell carcinoma (OSCC).^[21]^ However, such prognostic factors can only be obtained by surgical exploration and subsequent histologic examination before surgery, and there is no reliable marker that provides correct data regarding the potentiality of delayed cervical lymph node metastasis, strategy for preventive neck dissection and prognosis.

The aim of this study was to determine the efficacy of preoperative SIR assessed by LMR, NLR, PLR, and IRB score for predicting disease-specific survival (DSS) and delayed cervical lymph node metastasis in patients with early-stage OSCC undergoing surgery-based treatment.

## 2. Materials and Methods

### 2.1. Study groups

We retrospectively reviewed a database containing medical records for 72 consecutive patients who underwent curative treatment for histologically confirmed early-stage OSCC between January 2006 and December 2012 at Niigata University Medical & Dental Hospital (Niigata, Japan). This analysis excluded the patients showed preoperative clinical findings of infection or systemic inflammatory conditions. Information on the patient’s clinicopathological features, laboratory data, and information on treatment strategies were obtained from clinical records. DSS rates and delayed cervical lymph node metastasis were evaluated with the cause of death or detection of cervical lymph node metastasis determined from clinical records. The retrospective design of this study was approved by the Ethical Review Committee of Niigata University Medical & Dental Hospital (Niigata, Japan). Research was conducted in accordance with the 1964 Declaration of Helsinki and its later amendments. All study participants were provided the opportunity to opt out.

### 2.2. Calculations of LMR, NLR, and PLR

Preoperative complete blood cell counts were retrospectively extracted from patient clinical records. All white blood cell and differential blood count were obtained within one week prior to surgery.

LMR was calculated by dividing the absolute lymphocyte count by the absolute monocyte count, NLR was calculated by dividing the absolute neutrophil count by the absolute lymphocyte count, and PLR was calculated by dividing the absolute platelet count by the absolute lymphocyte count.

### 2.3. Cutoff values of LMR, NLR, and PLR

Receiver operating characteristic curve analysis was used to determine the cutoff values for LMR, NLR, and PLR using the statistical software IBM SPSS Statistics (version 20 for Windows; IBM Corporation, Armonk, NY). Optimal cutoff values for DSS and delayed cervical lymph node metastasis were determined by using receiver operating characteristic (ROC) curves and Youden’s Index.

### 2.4. IRB score

IRB score was determined as follows: high LMR, high NLR, and low PLR, which were each rated as 1, with all other values rated as 0. These scores were added to obtain IRB score (range: 0–3).

### 2.5. Statistical analysis

The end points were set as death from cancer and delayed cervical lymph node metastasis, and they were examined statistically by the log-rank test as univariate analysis and by the Cox proportional hazard model as multivariate analysis. Potential prognostic factors included gender, age, primary site, pathological T classification, histological grading (WHO), tumor depth, mode of invasion (YK classification^[22]^), LMR, NLR, PLR, and IRB score. All statistical analyses were conducted using the IBM SPSS Statistics (version 20 for Windows; IBM Corporation) software. A *P* value of <.05 was considered statistically significant.

## 3. Results

The mean follow-up period was 71.8 months (range: 1–148 months). The patients included 33 males and 34 females, and the mean age of the patients was 68.2 years (range: 26–92 years). The primary sites were the tongue in 33 cases, gingiva mandible in 14 cases, buccal mucosa in 9 cases, gingiva maxilla in 6 cases, floor of mouth in 2 cases, with hard palate in 2 cases, and lip in one case. The cases were classified according to the American Joint Committee on Cancer 8th edition TNM staging system: 24 cases (35.8%) were classified as T1 and 43 cases (64.2%) were classified as T2. The other patient records are summarized in Table 1. At the last follow-up, 50 patients had no evidence of disease status and 7 patients had died. Delayed cervical lymph node metastasis occurred in 16 patients. All of the patients in whom delayed cervical lymph node metastasis occurred were treated by neck dissection. The 5-year DSS rate in all patients was 87.3%, and the 5-year cervical metastasis-free survival rate was 75.5%.

**Table 1:** Prognostic factors for disease-specific survival in 67 patients with oral early squamous cell carcinoma.

Variables		Number of patients	5-yr survival rate (%)	*P* value
Gender	Male	33	96.9	.046
	Female	34	82.9	
Age	<72	35	94.3	.302
	≥72	32	83.3	
Primary site	Tongue	33	93.4	.230
	Gingiva mandible	14	85.1	
	Buccal mucosa	9	100	
	Gingiva maxilla	6	100	
	Floor of mouth	2	50.0	
	Hard palate	2	75.0	
	Lip	1	50.0	
T classification	T1	24	100	.060
	T2	43	85.5	
Histological grading	Grade 1	64	91.3	.223
	Grade 2	3	66.7	
Tumor depth	<5 mm	45	92.1	.666
	≥5 mm	22	86.4	
Mode of invasion (YK classification)	1, 2, 3	49	97.9	.003
	4C, 4D	18	68.2	
LMR	1, 2, 3	28	96.2	.121
	Low	39	85.5	
NLR	Low	17	100	.107
	High	50	86.6	
PLR	Low	34	96.9	.051
	High	33	83.2	
IRB score	Low (0, 1)	24	100	.041
	High (2, 3)	43	84.5	

IRB = inflammatory response biomarker, LMR = lymphocyte-to-monocyte ratio, NLR = neutrophil-to-lymphocyte ratio, PLR = platelet-to-lymphocyte ratio.

### 3.1. Cutoff values of LMR, NLR, and PLR

For LMR, the area under the curve (AUC) and optimal cutoff value for prediction of DSS were 0.598 and 6.41, respectively, with a sensitivity of 45.0% and a specificity of 85.7%. The AUC and optimal cutoff value for prediction of delayed cervical lymph node metastasis were 0.538 and 2.35, respectively, with a sensitivity of 87.5% and a specificity of 0%. For NLR, the AUC and optimal cutoff value for prediction of DSS were 0.600 and 1.47, respectively, with a sensitivity of 100% and a specificity of 28.3%. The AUC and optimal cutoff value for prediction of delayed cervical lymph node metastasis were 0.528 and 5.37, respectively, with a sensitivity of 18.8% and a specificity of 100%. For PLR, the AUC and optimal cutoff value for prediction of DSS were 0.626 and 135.3, respectively, with a sensitivity of 85.7% and a specificity of 56.7%. The AUC and optimal cutoff value for prediction of delayed cervical lymph node metastasis were 0.586 and 138.1, respectively, with a sensitivity of 68.8% and a specificity of 64.7%. Measurements exceeding the optimal cutoff values were regarded as being high.

### 3.2. Prognostic factors for DSS

**Figure 1.**
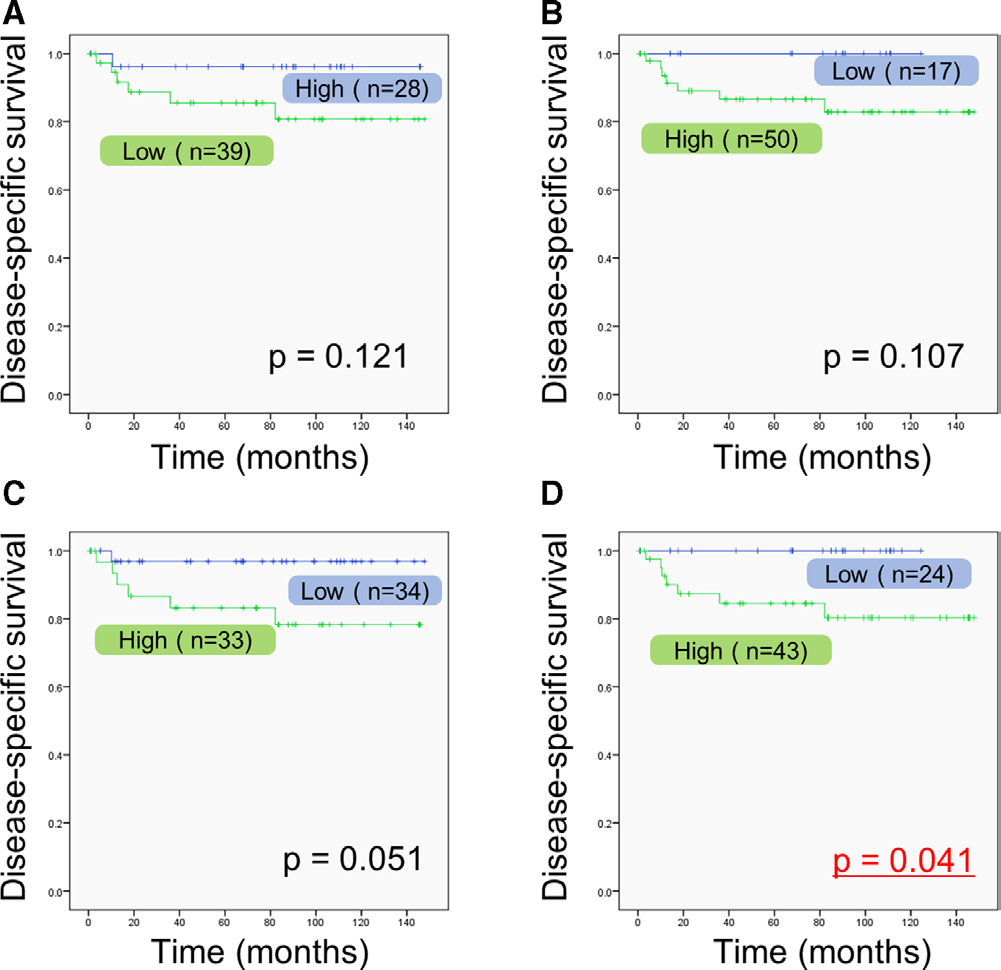
Kaplan–Meier survival curves of disease-specific survival according to inflammatory response biomarkers. (A) Lymphocyte-to-monocyte ratio, (B) neutrophil-to-lymphocyte ratio, (C) platelet-to-lymphocyte ratio, and (D) inflammatory response biomarker score.

From univariate analysis, gender (*P* < .05), poor mode of invasion (YK-4C and -4D) (*P* < .01), and high IRB score of 2 to 3 (*P* < .05) were identified as significant risk factors for DSS (Table 1). Cases with low LMR (*P* = .121; Fig. 1A), cases with high NLR (*P* = .107; Fig. 1B), and cases with high PLR (*P* = .051; Fig. 1C) showed a slight tendency for poorer prognosis, but the tendency was not statistically significant. Conversely, cases with high IRB score had a significant tendency for poor prognosis (*P* = .041; Fig. 1D). On the other hand, there were no independent factors for poor prognosis in the multivariate Cox proportional hazard model.

### 3.3. Risk factors for delayed cervical lymph node metastasis

**Figure 2.**
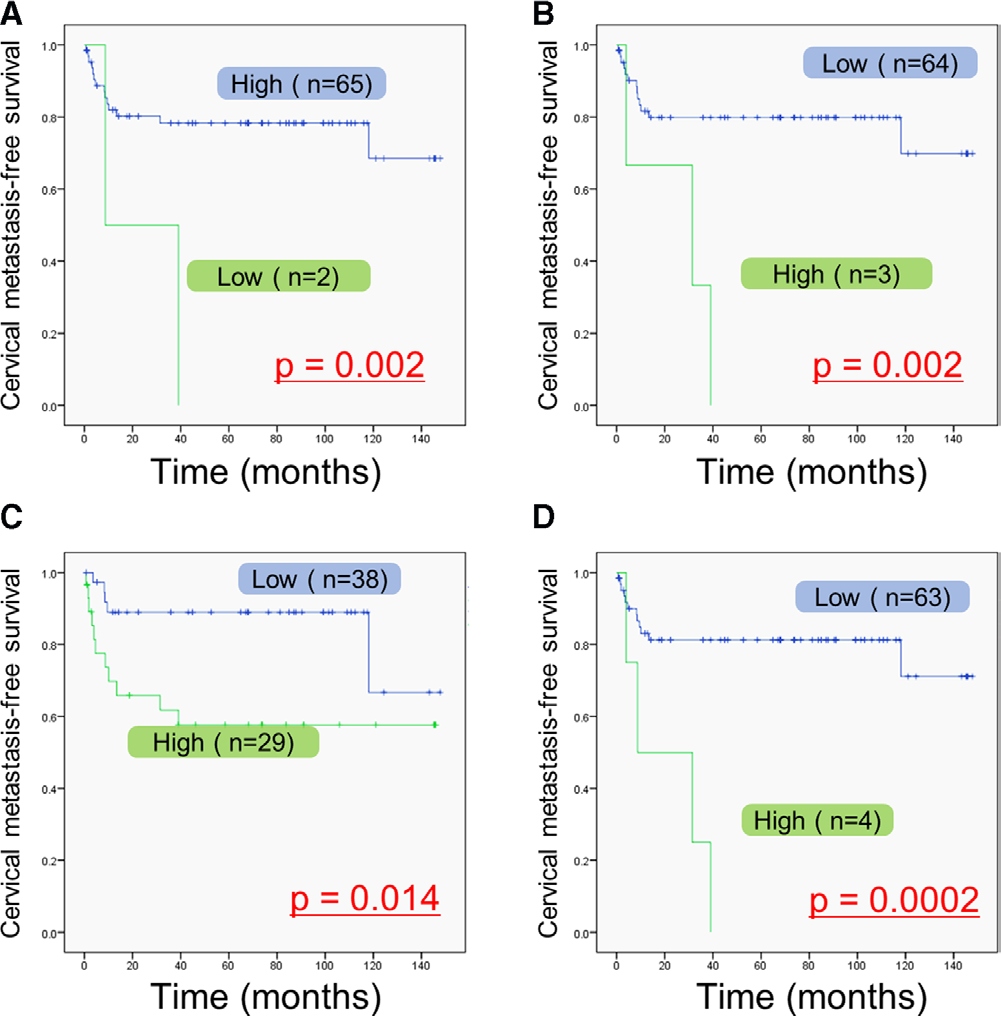
Kaplan–Meier survival curves of delayed cervical lymph node metastasis according to inflammatory response biomarkers. (A) Lymphocyte-to-monocyte ratio, (B) neutrophil-to-lymphocyte ratio, (C) platelet-to-lymphocyte ratio, and (D) inflammatory response biomarker score.

From univariate analysis, poor mode of invasion (YK-4C and -4D) (*P* < .01), low LMR (*P* < .05), high NLR (*P* < .01), high PLR (*P* < .05), and high IRB score (*P* < .01) were identified as significant risk factors for delayed cervical lymph node metastasis (Table 2). Cases with low LMR (*P* = .002; Fig. 2A), cases with high NLR (*P* = .002; Fig. 2B), and cases with high PLR (*P* = .014; Fig. 2C) showed a tendency for poorer prognosis. Furthermore, cases with high IRB score (*P* = .0002; Fig. 2D) obviously showed a tendency for poorer prognosis for delayed cervical lymph node metastasis. In the multivariate Cox proportional hazard model, poor mode of invasion (hazard ratio: 7.239, 95% confidence interval: 2.435–21.521; *P* = .0004), and high IRB score (hazard ratio: 4.885, 95% confidence interval: 1.525–15.651; *P* = .0076) were identified as independent risk factors for delayed cervical lymph node metastasis (Table 3).

**Table 2:** Prognostic factors for delayed cervical lymph node metastasis in 67 patients with oral early squamous cell carcinoma.

Variables		Number of patients	5-yr survival rate (%)	*P* Value
Gender	Male	33	80.1	.245
	Female	34	70.4	
Age	<72	35	71.4	.383
	≥72	32	82.3	
Primary site	Tongue	33	74.1	.716
	Gingiva mandible	14	60.6	
	Buccal mucosa	9	85.7	
	Gingiva maxilla	6	100	
	Floor of mouth	2	100	
	Hard palate	2	50.0	
	Lip	1	100	
T classification	T1	24	82.3	.328
	T2	43	71.6	
Histological grading	Grade 1	64	75.9	.583
	Grade 2	3	66.7	
Tumor depth	<5 mm	45	81.5	.075
	≥5 mm	22	63.6	
Mode of invasion (YK classification)	1, 2, 3	49	88.9	.00001
	4C, 4D	18	37.8	
LMR	1, 2, 3	65	78.3	.019
	Low	2	0	
NLR	Low	64	79.9	.002
	High	3	0	
PLR	Low	38	89.0	.014
	High	29	57.6	
IRB score	Low (0, 1)	63	81.3	.0002
	High (2, 3)	4	0	

IRB = inflammatory response biomarker, LMR = lymphocyte-to-monocyte ratio, NLR = neutrophil-to-lymphocyte ratio, PLR = platelet-to-lymphocyte ratio.

**Table 3:** Multivariate analysis of factors associated with delayed cervical lymph node metastasis.

Variables	Multivariate		
	Hazard ratio	95% confidence interval	*P* value
Mode of invasion			
4C, 4D vs 1, 2, 3	7.239	2.435–21.521	.0004
IRB score			
2, 3 vs 0, 1	4.885	1.525–15.651	.0076

IRB = inflammatory response biomarker.

## 4. Discussion

In this study, we analyzed the prognostic significance of LMR, NLR, PLR, and IRB score in early-stage OSCC patients who underwent surgery-based treatment with curative intent. Although the group of patients with low LMR, high NLR, and high PLR tended to have a poor prognosis for DSS, it was not significant in the univariate analysis. On the other hand, DSS in the group of patients with a high IRB score was significantly decreased in univariate analysis. However, no significant independent variable was found in multivariate analysis with all parameters. In this study, as described above, there was no evidence of a definite prognostic factor for DSS in patients with early-stage OSCC, unlike in past reports.^[15,17]^ The reason may be the limitation of early-stage OSCC. It should be noted here that these biomarkers, especially IRB score, may be useful for predicting delayed cervical lymph node metastasis. Abbate et al^[23]^ reported that NLR was a predictor for occult cervical metastasis in patients with early squamous cell carcinoma of the tongue. Park et al^[24]^ reported that patients with a high IRB score showed poorer DSS that that for patients with a low IRB score in OSCC patients who underwent surgery-based treatment.

It is well recognized that the presence of cervical lymph node metastasis is the most important prognostic factor in OSCC. Predicting potential lymph node metastasis in early-stage OSCC is very important for the therapeutic strategy. Various techniques have been used in order to more accurately identify cervical lymph node metastases clinically. However, there is no imaging device that can detect micrometastatic lymph nodes, and develop delayed cervical lymph node metastasis occurs in some patients who have negative imaging results. Previous studies have shown various risk factors of cervical lymph node metastasis such as histological grading,^[25–27]^ tumor thickness or depth,^[25,26,28–33]^ mode of invasion,^[26,29,30]^ perineural infiltration,^[26,33]^ angiolymphatic invasion,^[34]^ and desmoplasia.^[33]^ However, most of these factors are difficult to detect before surgery because surgical specimen is required, and we would have to say that it is not useful for considering the initial therapeutic strategy, particularly the indication for elective neck dissection (END).

On the other hand, several studies have suggested risk factors that may be useful for considering the initial therapeutic strategy. Hayashi et al^[28]^ showed that patients with stage I/II tongue carcinoma, which is more than 5 mm thick in initial US, are more likely to develop lymph node metastasis. Chien et al^[35]^ showed that the expression of CD105 and expression of VEGF in biopsy specimens from patients with early-stage oral cancer are valuable biomarkers for predicting cervical metastasis. The results of the present study, we had implied that IRB score may also be useful for predicting delayed cervical lymph node metastasis. These biomarkers may be useful for clinicians to stratify patients based on risks of lymph node metastatic for which END might be indicated. However, further studies with larger series of patients are needed to confirm assert the results and to establish cutoff values for recommending END in a clinically negative neck.

Although LMR, NLR, and PLR can predict the prognosis of patients with various types of cancer, their prognostic value and optimal cutoff points in patients with OSCC remain to be determined. In this study, ROC curves were analyzed to determine the optimal cutoff values for LMR, NLR, and PLR for prediction of DSS and delayed cervical lymph node metastasis. The optimal cutoff values were 6.41, 1.47, and 135.3 for LMR, NLR, and PLR, respectively, for prediction of DSS, and they were 2.35, 5.37, and 138.1 for LMR, NLR, and PLR, respectively, for prediction of delayed cervical lymph node metastasis. Abbate et al^[23]^ determined the cutoff value of NLR to be 2.93 and Wu et al^[17]^ determined the value to be 2.95 for lymph node metastasis in early-stage (cT1/T2N0) tongue cancer. Although the point using ROC curve to determine the cutoff value is the same, there is great difference from ours. One of the reasons for this may be the effect of targeting various primary sites in this study. As mentioned above, it would be clinically inconvenient since the cutoff values are determined according to the difference in the end points respectively. Furthermore, the AUC of each ROC curve is narrow, which is not a good blood examination method. The limitations of this study include its retrospective, single-institution design and the small sample number of patients. Large prospective randomized controlled trials are required to confirm our preliminary findings. However, despite these limitations, this study has demonstrated that the preoperative IRB score is a potential independent risk factor for delayed cervical lymph node metastasis in patients with early-stage OSCC. The IRB score can be obtained easily and it might be useful for risk stratification and establishment of a therapeutic strategy in patients with early-stage OSCC. Further investigation is needed to clarify the relationship between cytokines, classical tumor markers, and IRB score. In addition, larger prospective studies are needed to elucidate the mechanisms underlying the relationship between IRB score and cervical lymph node metastasis in patients with OSCC.

## Author contributions

**Conceptualization:** Toshihiko Mikami.

**Data curation:** Toshihiko Mikami, Wataru Katagiri.

**Formal analysis:** Wataru Katagiri.

**Supervision:** Akinori Funayama, Tadaharu Kobayashi.

**Validation:** Akinori Funayama, Kanae Niimi, Kenta Haga, Masami Kawaharada, Wataru Katagiri, Tadaharu Kobayashi.

**Visualization:** Toshihiko Mikami.

**Writing – original draft:** Toshihiko Mikami, Akihiko Nakamura.

**Writing – review & editing:** Akinori Funayama, Kanae Niimi, Wataru Katagiri, Tadaharu Kobayashi.
